# 

**Published:** 1877-02

**Authors:** 


### BOOKS AND PAMPHLETS RECEIVED.

The Electric Bath, its medical uses, effects and appliances. By G. M. Schweig, M.D., etc.

Proceedings of the Connecticut Medical Society, 1876.

A Report on Dermatology. By L. P. Yandell, Jr., M.D., 1876.

Fracture of the Patella, and treatment by a new method. By H. O. Marcy, A.M., M.D., 1876.

Spinal Irritation ; its pathology and treatment. By W. A. Hammond, M.D., etc. No. X., Vol. II., of "American Clinical Lectures."

Notes on the Burning of Theatres and Public Halls. Reflections on some of the causes of the great mortality occasionally attending such fires, and suggestions for improved security to life. The antiquity of the drama and the opening of theatres in America, with a chronological list of theatres and other public edifices burned. By J. M. Toner, M.D. 1876.

Walsh's Physician's Combined Call-book and Tablet. Second edition.

The Physician's Hand Book for 1877. By W. Elmer, M.D., and A. D. Elmer, M.D.

The Hypertrophied Prostate. By R. A. Weir, M.D., etc.

Diagnosis of those Diseases of the Eye which can be seen without the Ophthalmoscope. By Henry D. Noyes, M.D., etc.

On Tracheotomy and Laryngotomy. By H. B. Sands, M.D., etc. Nos. VIII., V., and VII., of Vol. II., of "A Series of American Clinical Lectures."

Annual Report of the Northern Hospital for the Insane, of the State of Wisconsin, for the year ending Sept. 30, 1876.

The Ovulation theory of Menstruation : Will it Stand? By A. Reeves Jackson, A.M., M.D., etc. New York : Wm. Wood & Co., 1876.

Micro-Photographs of Human Blood Discs ; of Epithelium lining the Lymph Sac of the Frog, and of the head of a cysticercus from the hare, magnified respectively 420, 300 and 40 diameters. Photographed by Dr. O. C. Oliver, of Chicago.

### ANNOUNCEMENTS FOR THE MONTH.

**MONDAYS.** SOCIETIES.

*Mondays, Feb.* 5 *and* 19.—Chicago Medical Society. Regular meetings.

*Mondays, Feb.* 12 *and* 26.—Chicago Soc. of Phys. and Surgeons. Regular meetings.

CLINICS. *Every Monday.*

At Eye and Ear Infirmary, 2 P. M.—Prof. Holmes.

At Central Dispensary (Wood and Harrison sts.)—2 P.M. *Gynecological*, Dr. Adolphus; 3 P.M. *Diseases of Children*, Dr. R. S. Hall.

At Mercy Hospital—2 P. M., *Surgical*, Prof. Andrews.

At Rush College—2¼ P. M., *Medical*, Dr. Bridge.

At Chicago College, 2 P. M *Gynecological*—Prof. Merriman.

LECTURES. *Every Monday.*

At Rush Medical College (Harrison and Wood sts.)—9½ to 12½ o'clock, Profs. Freer, Lyman and Powell; 4 to 6, Profs. Gunn and Haines. At Chicago College—8½ to 12½, Profs. Jewell, Hyde, Hatfield and Bond; 3 to 6, Profs. Nelson and Davis, Quine and Andrews, and Roler. At Woman's College—8½ to 11½, Profs. Hotz, Marguerat and Earle; 3 to 5, Profs. Paoli and Stevenson.

**TUESDAYS.** SOCIETIES.

*Tuesday, Feb.* 13.—Academy of Science. Regular meeting at 8 P. M. (263 Wabash av.)

CLINICS. *Every Tuesday.*

At Eye and Ear Infirmary—2 P. M., Prof. Jones.

At County Hospital—2 P. M., *Medical*, Prof. Bevan. 3 P. M., *Surgical*, Prof. Bogue.

At Mercy Hospital—2 P. M., *Medical*, Prof. Hollister.

At Chicago College—2 P. M., *Gynecological*, Prof. Roler.

At Central Dispensary—2, *Surgical*, Dr. Graham.

LECTURES. *Every Tuesday.*

At Rush College—9½ to 12½, Profs. Miller, Allen and Parkes; 4 to 6, Profs. Gunn and Haines. At Chicago College—8½ to 12½, Profs. Jewell, Quine, Merriman and Bond; 3 to 6, Profs. Johnson and Nelson, Andrews and Hatfield, and Byford. At Woman's College—9 to 12, Profs. Bartlett, Curtis and Fitch; 4 to 6, Profs. Hayes and Macdonald.

**WEDNESDAYS.** CLINICS. *Every Wednesday.*

At Eye and Ear Infirmary—2 P.M., Prof. Holmes.

At County Hospital—2 P. M., *Gynecological*, Prof. Fitch; 2 P. M., *Ophthalmological*, Dr. Montgomery.

At Chicago College—2 P. M., *Gynecological*, Prof. Nelson.

At Mercy Hospital—2 P. M., *Ophthalmological*, Prof. Jones.

At Central Dispensary—3 P. M., *Dermatological*, Dr. Maynard.

LECTURES. *Every Wednesday.*

At Rush College—9½ to 12½, Profs. Freer, Allen and Parkes; 4 to 6, Profs. Etheridge and Haines. At Chicago College—8½ to 12½, Profs. Hyde, Isham, Hatfield and Bond; 3 to 6, Profs. Davis and Curtis, Quine and Jones, and Roler. At Woman's College—8:30 to 10:30, Profs. Hotz and Marguerat; 11, Prof. Blake; 3 to 6, Profs. Paoli, Graham and Stevenson.

**THURSDAYS.** CLINICS. *Every Thursday.*

At Eye and Ear Infirmary—2 P. M., Prof. Hotz.

At Mercy Hospital—2 P. M., *Medical*, Prof. Davis.

At Rush College—2 P. M., *Medical*, Prof. Ross; 3 P. M., *Diseases of the Nervous System*, Prof. Lyman.

At Chicago College—2 P. M., *Gynecological*, Prof. Merriman.

At Central Dispensary—2 P.M., *Surgical*, Dr. Graham.

LECTURES. *Every Thursday.*

At Rush College—9½ to 12½, Profs. Miller, Allen and Parkes; 4 to 6, Profs. Gunn and Etheridge. At Chicago College—8½ to 12½, Profs. Hollister, Isham, Merriman and Bond; 3 to 6, Profs. Johnson and Nelson, Andrews and Hatfield, and Byford. At Woman's College—9 to 12, Profs. Bartlett, Curtis and Fitch; 4 to 6, Profs. Thompson and Macdonald.

**FRIDAYS.** SOCIETIES.

*Friday, Feb.* 9.—State Microscopical Society of Illinois. Regular meeting, 8 P. M.

CLINICS. *Every Friday.*

At County Hospital—2 P. M., *Medical*, Prof. Bevan; 3 P. M., *Surgical*, Prof. Bogue.

At Mercy Hospital—2 P. M., *Medical*, Prof. Davis.

At Chicago College—2 P. M., *Gynecological*, Prof. Roler.

At Central Dispensary—2 P. M., *Gynecological*, Dr. Adolphus; 3 P.M., *Diseases of Children*, Dr. R. S. Hall.

LECTURES. *Every Friday.*

At Rush College—9½ to 12½, Profs. Freer, Allen and Parkes; 4 to 6, Profs. Gunn and Holmes. At Chicago College—8½ to 12½, Profs. Hollister, Isham, Hatfield and Bond; 3 to 6. Profs. Davis and Curtis, Jones and Quine, and Roler. At Woman's College—9 to 12, Profs. Dyas, Marguerat and Earle; 3 to 5, Prof. Hayes; 5, Prof. Stevenson.

**SATURDAYS.** CLINICS. *Every Saturday.*

At Chicago College—2 P. M., *Surgical*, Prof. Andrews or Isham; *Gynecological*, Prof. Nelson; 3 P. M., *Medical*, Prof. Johnson.

At Rush College—2 P. M., *Surgical*, Prof. Gunn.

LECTURES. *Every Saturday.*

At Rush College—8½ to 12½, Profs. Lyman, Miller, Etheridge and Parks. At Chicago College, 8½ to 11½, Profs. Hollister, Quine and Hyde; 3 to 6, Profs. Nelson and Johnson, Andrews and Quine and Byford. At Woman's College—9 to 12, Profs. Bartlett, Earle and Fitch; 3 to 6, Profs. Paoli, Thompson and Macdonald.

At all the above named Clinics visiting regular practitioners are, we believe, admitted.

At the South Side Dispensary (Chicago College) there are six daily special Clinics, for sections of the classes of the Chicago College.

